# Genome Sequence of the Symbiotic Type Strain Mesorhizobium helmanticense CSLC115N Isolated from Lotus corniculatus Nodules

**DOI:** 10.1128/genomeA.00412-18

**Published:** 2018-05-10

**Authors:** Analía I. Sannazzaro, Gonzalo A. Torres Tejerizo, Marina Caballero, Diana Dip, Mariano Pistorio, María Julia Estrella

**Affiliations:** a INTECH (Instituto Tecnológico Chascomús), CCT-La Plata, CONICET-Universidad Nacional de San Martín, Chascomús, Argentina; b IBBM (Instituto de Biotecnología y Biología Molecular), CCT-La Plata, CONICET, Departamento de Ciencias Biológicas, Facultad de Ciencias Exactas, Universidad Nacional de La Plata, La Plata, Argentina

## Abstract

Mesorhizobium helmanticense is a novel species that was isolated from root nodules of Lotus corniculatus grown in an alfisol soil from Carbajosa de la Sagrada, a Mediterranean region in the province of Salamanca in northwest Spain. The whole-genome sequence of the type strain M. helmanticense CSLC115N is reported in this study.

## GENOME ANNOUNCEMENT

The need to introduce sustainable agricultural practices around the world has revitalized an interest in the study of the genetic diversity of microorganisms that establish beneficial interactions with plants, including rhizobia, which are symbiotic nitrogen-fixing bacteria that are beneficial for legumes (1). Different studies have been carried out in recent years to understand better the phylogenetic diversity of symbionts that interact with the legume Lotus corniculatus, a perennial species that has a natural distribution in Western Europe and North Africa and that is of great economic importance for the production of pastures and silage in different regions of the world (2–4).

The rhizobia of L. corniculatus cultivated in an alfisol soil of Carbajosa de la Sagrada (Salamanca, Spain), had been characterized. The results revealed a high phylogenetic diversity of L. corniculatus endosymbionts, with most isolates belonging to previously described Mesorhizobium species (5). However, some strains were later found to represent a novel species, for which the name Mesorhizobium helmanticense was proposed (6). With the aim of deepening the knowledge of the functions that characterize this new species belonging to the Mesorhizobium genus, in this work we sequenced the type strain of M. helmanticense, CSLC115N.

The M. helmanticense LMG 29734^T^ (= CSLC115N^T^) strain was taken from the BCCM/LMG Bacteria Collection. Genomic DNA was isolated using a Maxwell 16-tissue DNA purification kit (cat. no. AS1030) and a Maxwell 16 instrument (cat. no. AS2000). A Quantus fluorometer was used to estimate the DNA concentration of the solution. Whole-genome sequencing analysis was performed by the Oxford Genomics Center (University of Oxford, United Kingdom). Paired-end (150-bp) sequence reads were generated using the Illumina HiSeq 4000 platform. Library preparation was performed using an in-house adapted protocol of the NEB prep kit.

In total, 7,816,693 sequence reads were obtained, yielding a total of 1,153,487,657 bp of sequence information. The Illumina reads were assembled by the GS *de novo* assembler software (gsAssembler, version 2.8, Roche) with the final outcome of 145 large contigs (>200 bp). The average contig size was 47,445 bp, with the largest being 379,735 bp. The estimated genome size is around 6.9 Mb, and, accordingly, the coverage obtained was approximately 167-fold. The genome features an average GC content of 62.44%. The genome was annotated applying the NCBI Prokaryotic Genome Annotation Pipeline, which predicted 6,430 protein-coding sequences, 261 pseudogenes, and 48 tRNAs.

### Accession number(s).

This whole-genome shotgun project has been deposited at DDBJ/ENA/GenBank under the accession number PZJX00000000. The version described in this paper is the first version, PZJX01000000.
